# Intolerance of uncertainty and decisions about delayed, probabilistic rewards: A replication and extension of Luhmann, C. C., Ishida, K., & Hajcak, G. (2011)

**DOI:** 10.1371/journal.pone.0256210

**Published:** 2021-09-24

**Authors:** Luis F. Ciria, María J. Quintero, Francisco J. López, David Luque, Pedro L. Cobos, Joaquín Morís

**Affiliations:** 1 Universidad de Granada, Granada, Spain; 2 Universidad de Málaga, Málaga, Spain; 3 Instituto de Investigación Biomédica de Málaga (IBIMA), Málaga, Spain; 4 Universidad Autónoma de Madrid, Madrid, Spain; Texas A&M University, UNITED STATES

## Abstract

Intolerance of Uncertainty (IU) is thought to lead to maladaptive behaviours and dysfunctional decision making, both in the clinical and healthy population. The seminal study reported by Luhmann and collaborators in 2011 [1] showed that IU was negatively associated with choosing a delayed, but more probable and valuable, reward over choosing an immediate, but less probable and valuable, reward. These findings have been widely disseminated across the field of personality and individual differences because of their relevance for the understanding of the role of IU in the development and maintenance of anxiety-related disorders. Given their importance it would be desirable to have replications of this study, but none have been carried out so far. The current study has been designed to replicate and extend Luhmann et al.’s results. Our sample will include 266 healthy participants (more than five times the sample size used by Luhmann et al.) to detect with a power of 95% the effect size that can be detected with a power of 33% in the original study. To increase our chances of getting such a sample size, the experiment will be conducted online, To increase our chances of getting such a sample size, the experiment will be conducted online, adding check trials to the original decision-making task to monitor participants’ engagement. Additionally, we will explore the role of impulsivity in the relationship between IU and willingness to wait. This study will add empirical evidence about the role of IU in decision making and, in case of replication of Luhmann et al.’s results, will support the hypothesis that high-IU individuals may engage in inefficient or costly behaviour in exchange for less time enduring an uncertain situation.

## Introduction

Intolerance of uncertainty (IU) has been defined as “an individual’s dispositional incapacity to endure the aversive response triggered by the perceived absence of salient, key, or sufficient information, and sustained by the associated perception of uncertainty” [2]. A distinctive feature of IU is that uncertainty involves a future-oriented unpredictable component, a hallmark that makes a difference with intolerance of ambiguity, which would involve unpredictability regarding the “here and now” [3]. Perceiving uncertainty about future relevant events has been claimed to be crucial to understand the relationship between IU and pathological anxiety, as this condition has been related to maladaptive anticipatory reactions to unpredictable future threats [4]. Consequently, it is not surprising that IU has been found to play a crucial role in several anxiety-related disorders [5–9], as well as a transdiagnostic vulnerability factor for the development and maintenance of anxiety and depression symptoms [7,10,11].

Studies about IU have greatly improved our knowledge about the concept itself, its assessment, its relationship with different mental disorders as well as with other dispositional factors [12]. However, there is insufficient knowledge about the expression of IU in terms of behaviour and decision-making [12]. Therefore, the assessment of IU has to rely mostly on self-report measures that may be more prone to subjective biases. Additionally, a better understanding about how IU relates to behaviour and decision-making is warranted if we are to find out the causal mechanisms through which IU promotes the development and maintenance of symptoms in different psychopathology conditions.

One extended idea about IU is that the incapacity to endure uncertainty in some individuals makes them engage in behaviours and decisions intended to turn uncertain situations into more predictable ones or to enhance perceived control [2,5,13,14]. Consistently, high IU individuals have been described as risk avoiders [2] who, if needed, may sometimes behave to gain a feeling of predictability even at the cost of efficiency [15–17]. However, Luhmann et al. [1] proposed that decision-making guided by IU may not always aim at avoiding risk or enhance perceived predictability. According to them, the motivation driving behaviour and decision-making in high IU people is the urgent need to escape from (or avoid) the distress caused by uncertainty. This hypothesis slightly differs from the previous one in that it explicitly states that the aversion to uncertainty-related distress is greater than the aversion to uncertainty itself. Interestingly, this idea has empirical implications. In some circumstances, high IU people may choose more uncertain outcomes of lower value to avoid, or escape from, longer periods of distress waiting for less uncertain outcomes of higher value.

Luhmann et al. [1] provided evidence supporting their hypothesis in an experiment with 50 non-clinical participants. They went through 100 trials in each of which they had to decide between selecting an immediate choice with a 50% chance of receiving 4 cents or waiting to select a delayed choice with a 70% chance of receiving 6 cents. In both cases, participants knew right after their response if they had obtained the reward or not. Luhmann et al. predicted that high IU participants, compared with low IU participants, would show a higher preference for the immediate but more uncertain and less valuable outcome over the delayed but less uncertain and more valuable outcome. Consistently, they found a negative association between IU scores and willingness to wait for the second stimulus, after controlling for trait anxiety (TA) and monetary delay discounting, as measured through Kirby and Marakovic’s Delay-Discounting questionnaire [18].

Luhmann et al.’s [1] study has been cited very often, as their results have important implications about the role of IU in some psychopathologies, and the conception of IU. However, as far as we know, Tanovic et al.’s study [19] is the only attempt to replicate Luhmann et al.’s results. Unfortunately, both the analyses conducted and the results found by Tanovic et al. [19] were considerably different from Luhmann et al.’s. First, Tanovic et al. only found a relationship between inhibitory IU (I-IU, one of the two factors of the IU scale) and willingness to wait, whereas Luhmann et al. did not include the IU factors in the analyses. Second, Tanovic et al. did not conduct any analysis to assess the specificity of the relationship found between I-IU and willingness to wait. Finally, Tanovic et al. used a sample size of 56 participants, which may explain the differences between their results and those found by Luhmann et al. In our study, we will report the results of a considerably strict replication and extension of Luhmann et al.’s study.

Our replication will differ from the original study in a few respects. First, the sample size will be much larger than the original study to be able to detect a much smaller effect. Second, it will be conducted as an online study instead of an experiment conducted in a laboratory to increase our chances of getting a large sample size. Additionally, the behavioural task will be slightly modified to monitor participants’ engagement and to have an adequate control of their performance. As in the original study, we will test if the referred association is observed after controlling for trait anxiety. Another important concern regarding the task used by Luhmann et al. [1] is that the ability to refrain from choosing immediate small rewards to get delayed and more valuable rewards may be strongly related to impulsivity [20]. Therefore, as Luhmann et al. [1], we will perform statistical analyses to test if the association between IU and willingness to wait is found after controlling for a delay discount factor calculated from participants’ responses in a questionnaire based on decisions between monetary rewards differing in magnitude and delay. To extend the original findings, participants in our study will fulfil the Spanish version of the short UPPS‑P impulsive behaviour scale (SUPPS-P) [21]. This way, we will be able to assess the specificity of the relationship between IU and willingness to wait after controlling also for impulsivity, in addition to the trait anxiety and delay discount.

## Method

### Participants

Undergraduate students from Spanish universities will take part in the experiment in exchange for a monetary reward and course credit. The amount of money earned will depend on their performance during the decision-making task itself. We will include males and females with normal or corrected-to-normal vision. Before being recruited to the study, participants will read and sign the informed consent. Participants will be naïve to the aim of the study in order to avoid expectation effects. Once all participants have completed the experiment, they will be debriefed on the purpose of the study. The experimental procedure has been approved by the Ethics Committees of both universities (CEUMA-46-2020-H; CEI-102-1940), complying with the Declaration of Helsinki [22].

### Questionnaires

Following Luhmann et al. [1], all participants will complete IU, TA, and delay discounting questionnaires. As discussed in the introduction section, an impulsivity test will also be included. Specifically, we will use the following questionnaires:

#### The Spanish adaptation of the Intolerance of Uncertainty Scale

The IUS [23,24] is a 27-item self-report measure that assesses the degree to which individuals find uncertainty to be distressing and undesirable (internal consistency of .91 and test-retest reliability of .78; [25]). The IUS includes two subscales known as Prospective Intolerance of Uncertainty (11-items) and Inhibitory Intolerance of Uncertainty (16-items). Items are rated on a five-point Likert scale ranging from 1 (*not at all characteristic of me*) to 5 (*extremely characteristic of me*).

#### The Spanish adaptation of the trait subscale of the State Trait Anxiety Inventory, Form Y

This subscale of the STAI [26,27] is a 20-item self-evaluation questionnaire with good psychometric properties (internal consistency between .90 and .95, and test-retest reliability between .84 and .91). While the STAI includes two subscales known as Trait Anxiety (20 items) and State Anxiety (20 items), participants will only complete the Trait Anxiety (TA) subscale. Items in the TA subscale are rated on a four-point Likert scale ranging from 0 (nothing) to 3 (a lot).

#### The Spanish adaptation of the Delay-Discounting Test [28,29]

This test is a 27-item monetary-choice questionnaire asking for individual preferences between smaller, immediate rewards and larger, delayed rewards varying on their value and time to be delivered (test-retest reliability between .63 and .77). After reading each item (e.g., “Would you prefer 55€ today, or 75€ in 61 days?”) participants have to indicate which alternative she or he would prefer to receive by marking the alternative in the questionnaire.

#### The Spanish adaptation of the SUPPS-P Impulsive Behavior Scale [21,30]

This test is a 20-item inventory designed to measure five distinct personality facets of impulsive behaviour: Positive urgency, negative urgency, lack of perseverance, lack of premeditation, and sensation seeking (internal consistency between .61 and .81). Items are rated on a four-point Likert scale ranging from 1 (strongly agree) to 4 (strongly disagree). Previous studies have shown weak correlations between the Delay-Discounting Test and different trait measures of impulsivity [28,31]. This indicates that this test might be capturing only some aspects of impulsivity. Because of this, it is expected that including SUPPS-P in the analysis, compared to using only the Delay-Discounting Test, will provide additional information, and allow a better interpretation of the results obtained (see below).

### Procedure

Participants will sign up through an online experiment database, and will complete informed consent, questionnaires, and a decision-making task online. Questionnaires will be completed online using Google Docs. The decision-making task will be coded in JavaScript using Psychopy (version 2020.1) [32] and jsPsych (version 5.0.3) [33], and will be hosted and deployed online on a secure server at University of Malaga. The task can be accessed at http://causal.uma.es/test_exp. They will be asked to use their personal computers (i.e., the task will not run in smartphones or tablets) in a semi-isolated dim-lit room, without breaks during the entire session. At the beginning of the session, they will be asked to shut down other applications running in parallel to avoid long page loading times and timing delays. They will be given the option to run the experiment onsite if they choose to do so.

**Fig 1:**
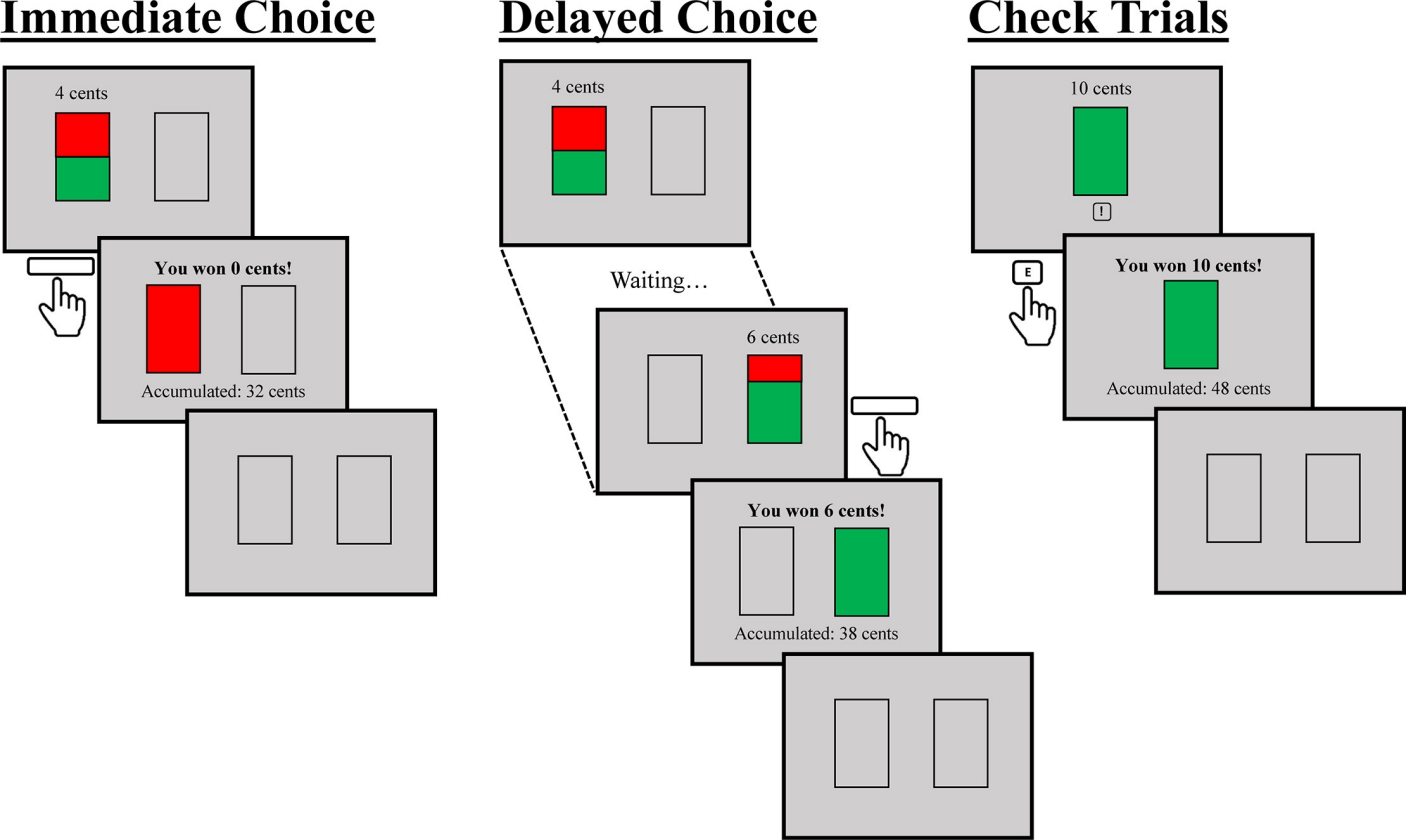
Sequence of the decision-making task. Left: Trial in which the immediate choice is selected, but no reward is won. Centre: Trial in which the delayed choice is selected, and the reward is won. Right: Check trial to monitor the participant’s engagement, in which an appropriate response is provided, and the reward is won.

Participants will be informed about the conditions of the study and will have to provide their consent to participate. Then, gender and age will be recorded, as well as a set of questionnaires about demographic data and personality traits will be completed online (see Material section for details). Participants will be instructed to read each question in the given order and not to skip questions or go back to an earlier one. After completing them, participants will receive a link that will redirect them to an online platform to perform the decision-making task described in Luhmann et al.’s study. In this task, each trial will begin with the presentation of two empty rectangles displayed on a grey background, side by side on the centre of the computer screen, for a minimum period of time of 0.5 s (see below). Then, the left rectangle will be filled in with two colours (red and green). The colours in the rectangles will provide information about the probability of being and not being rewarded (see Fig 1), which is represented by the size of the green and red areas, respectively (e.g., if both areas have the same size, the likelihood of receiving the reward is 50%). Simultaneously, the monetary value of the reward will be displayed above the rectangle. Selecting this first option (i.e., the immediate choice) will always lead to a 50% chance of receiving a 4 cents reward. Alternatively, participants can wait for the appearance of a delayed choice indicated by the display of the green and red areas in the right rectangle and the offset of these colours in the left rectangle. This delayed choice will always lead to a 70% chance of receiving 6 cents. Immediately after participants select any choice by pressing the spacebar, the rectangle will be completely filled in for 1000 ms with only one colour, green or red, indicating whether or not, respectively, the reward has been gained. Participants will also receive additional information through text messages telling whether they received the reward or not, and the money accumulated so far. The duration of the feedback will be 1 s. To prevent large deviations from the probabilities described to the participants, the task will be pseudorandom. In each block of ten choices of the same type, the number of wins and losses will be fixed (5 and 5 in the case of immediate choices, and 7 and 3 in the case of delayed choices), and their order randomized.

Due to the online nature of this study, a new type of trial will be added. Ten check trials will be included to monitor the engagement of participants through the decision-making task. In these trials, the only choice will be a 100% chance of receiving 10 cents, which will be indicated by the display of a new rectangle completely filled in with green colour at the centre of the screen. Participants will be allowed to select this choice by pressing key “E” on their keyboard within three seconds after the rectangle onset. If this choice is not selected within three seconds, or the space bar is pressed, a message will be displayed telling participants that they have missed the possibility of gaining 10 cents. Note that they will have to press a different key (key “E”) on check trials in order to detect automatic or inattentive performance throughout the task. Each check trial will pseudo-randomly appear among the last 5 trials of each 10-trial block. This new type of trials constitutes a modification of the original task but given that participants will be able to complete the task online, it will provide a chance to remove participants that are not adequately paying attention to the task.

A critical detail of the task is that the delay between the onset of the immediate choice and the delayed choice will vary between 5 and 20 seconds according to a truncated exponential distribution for preventing participants from knowing how long they will have to wait for the second choice. Crucially, participants will be explicitly told that they will not be able to move on to the next trial any sooner by selecting the immediate choice since this action will simply extend the following intertrial interval (ITI). The ITI will follow the same variable time schedule as the delays between choice options. Thus, if in a given trial the programmed delay between choice options is, for instance, 11 s, and the participant takes 2 s to select the first choice, the next ITI will be 9.5 s. It would be the result of adding the 11 s of programmed delay minus the 2 s response time to the minimum ITI duration of 0.5 s. Participants will complete 10 practice trials to familiarize with the task procedure. In these trials, they will be instructed to make specific choices (i.e., on half of the practice trials, they will be directed to select the immediate choice, while on the other half, they will be directed to select the delayed choice) to ensure that they are exposed to the full range of possible outcomes. Two additional practice trials will also be added to get participants familiar with the check trials. In both of them, they will be instructed to press key “E” within three seconds. After practice trials, a total of 100 trials (plus 10 check trials) will be presented. The task will last for approximately 25 minutes.

Participants not completing the entire session and the questionnaires will be excluded from analyses. In addition, participants responding incorrectly on more than two check trials, with more than 10% of reaction times (RTs) below 200 ms when choosing the immediate choice, more than 10% of RTs greater than 3000 ms when choosing the delayed choice, or more than 20 responses to space bar or key "E" during ITI, will be excluded from analyses.

### Planned sample size

To calculate the sample size, we created an R [34] script (https://osf.io/va5db/) that uses the packages pwr (version 1.3–0) [35] and MBESS (version 4.6.0) [36,37]. Following the small-telescopes approach proposed by Simonsohn [38], we estimated a target effect size based on the original experiment. According to Simonsohn’s proposal, the target effect size would be the effect size that can be detected with a power of 33% in the original study. In Luhmann et al.’s study, the main results were obtained from a multiple regression analysis where IU and TA scores, and delay discount factor were included as predictors, and the percentage of trials in which a delayed choice was made, p(Wait), was included as the dependent variable. This analysis yielded significant regression coefficients for IU and delay discount, but not for TA. Based on this result, we considered two possible sample sizes to choose the most conservative one. In the original study, that multiple regression model had 3 degrees of freedom in the numerator and 45 in the denominator. For the omnibus regression, the effect size that could be detected with a 33% power in the original study is f^2^ = 0.081. The sample size required to detect that effect size in a multiple regression with a power of 95%, using an α = .05, would be of 215 participants. However, the main theoretical point is the relation between IU and the behavioural task, as measured by p(Wait). Focusing on the targeted correlation coefficient between IU and p(Wait), the effect size that can be detected with a 33% power in the original study is f^2^ = 0.049, and the sample size required to detect this effect with a power of 95% would be n = 266.

As mentioned before, we chose the most conservative sample size, n = 266. This sample would be 5.4 times the original sample, and lead to a highly powered experiment. Given that the recruitment will be carried out in batches, recruitment will stop as soon as at least 266 participants complete the task and match the data selection criteria described in the methods section.

### Statistical analysis

The analyses that will be carried out are derived directly from Luhmann et al. The R script with all of the analyses described are already available at https://osf.io/b8hfc/. The packages lm.beta (version 1.5–1) [39], papaja (version 0.1.0.9942) [40], patchwork (version 1.0.0) [41] and tidyverse (version 1.3.0) [42] are used in this script.

We will report the descriptives and zero-order correlations of the variables probability of waiting in the behavioural task [p(Wait)], IUS score, TA score, and discount factor [(1/1+k), DELAY-DISCOUNT]. We will add the descriptives and correlations also of the SUPPS-P Impulsive Behavior Scale. As in the case of Luhmann et al., the main analysis will be a hierarchical linear regression (see Table 1). Using p(Wait) as the dependent variable, two models will be considered: The first one will include TA and DELAY-DISCOUNT as predictors (Model 1), and the second model will include IU as an additional predictor (Model 2). Extending Luhmann et al.’s study, an additional hierarchical linear regression analysis will be conducted. The Model 1, that has TA and DELAY-DISCOUNT as predictors will be compared with a model that also includes SSPPS-P as predictor (Model 3), and this model will be compared with the final model that also adds IU (Model 4). In both hierarchical regression analyses, the difference between the models will be tested using an ANOVA.

**Table 1:** Summary of the regression models.

Models	Predictors	Compared with
Model 1	TA, DELAY-DISCOUNT	
Model 2	TA, DELAY-DISCOUNT, IU	Model 1
Model 3	TA, DELAY-DISCOUNT, SUPPS-P	Model 1
Model 4	TA, DELAY-DISCOUNT, SUPPS-P, IU	Model 3

The comparison between Model 1 and Model 2 will indicate if there is a relationship between IU and p(Wait) after controlling for TA and DELAY-DISCOUNT scores. This is the same comparison that was carried out by Luhmann et al. (2011) [1]. A positive finding in this comparison would replicate their original results. The comparison between Model 1 and Model 3 tests a possible relationship between SUPPS-P and p(Wait) after controlling for TA and DELAY-DISCOUNT scores. Finally, the comparison between Model 3 and Model 4 allows to check if the relationship between IU and p(Wait) is observed after controlling, additionally, for SUPPS-U. These two final comparisons are an extension of the work of Luhmann et al., as described before.

As in Luhmann et al., the association between the same predictors and the median reaction time in trials with an immediate choice will be tested with the same hierarchical regression analyses just described using the median response time in trials with an immediate choice as the dependent variable. This summary will be calculated using the reaction time of all the immediate choice trials of the original condition of the behavioural task (i.e., excluding the check trials).

Two diagnostic plots will be included for each model: a scatterplot of the residuals and predicted values, and a Q-Q plot. If the diagnostic plots show that the linear regression assumptions are not met, a robust linear regression technique (MM-estimates, as implemented by the MASS package in R) will be used.

Two variables will be calculated considering the proportion of delayed choices. The first one will be the proportion of delayed choices when the previous trial was a nonreinforced, delayed-choice trial. The second one, the proportion of delayed choices after any other type of standard trial (i.e., trials after a checking trial will be ignored). As in Luhmann et al., a paired t-test between these two variables will be calculated, as well as the Pearson correlation between the difference of these two proportions.

### Exploratory analysis

Exploratory analyses [43,44] will be carried out to separately study the role of the different factors of the SUPPS-P scale [45], as well as the prospective and the inhibitory factors of IU scale [46]. We have no specific hypothesis regarding each subscale and factor, and all statistical analysis will be performed accordingly.

### Data collection and storage

Data collection is expected to begin in mid-November, 2021. The behavioural data and questionnaire scores will be available at the OSF repository at https://osf.io/qyk87/?view_only=556eed2f698b472ca6767e763f768e16, with unrestricted access, as soon as the data collection has been completed. The data will be completely anonymized, and the original data with identifiers will be deleted. The R script with all of the analyses described are already available at https://osf.io/b8hfc/.

## Implications of the expected results

The consideration of IU as a main source of maladaptive and inefficient behaviour and decision making that may severely affect people suffering from anxiety-related mental disorders has been widely postulated in the literature, but this claim lacks empirical support [2]. As far as we know, Luhmann et al.’s study [1] provides the clearest evidence showing that people scoring high in IU tend to choose options that are riskier and less valuable as long as they imply less time waiting in an uncertain situation. This strongly suggests that many examples of costly behaviour in anxiety-related psychopathologies, such as excessive avoidance, may be understood as instances of decisions aimed to avoid time enduring uncertainty [see also 13,14]. Moreover, Luhmann et al.’s results also suggest that the same mechanism may underlie many instances of high-cost decision making in the non-clinical population. The results of our proposed study will be informative whether a significant association between IU and willingness to wait is found or not. In the former case, Luhmann et al.’s results will have been replicated, at least in essence, with a much larger sample, and with additional analyses that will refine our understanding of the relationship between IU and willingness to wait. In the latter case, our results may indicate that there is an underdetermination of the original hypothesis that would need to be taken into account in order to better describe the relation between IU and the behavioural task used by Lumann et al.
